# Epidemiological situation of yaws in the Americas: A systematic review in the context of a regional elimination goal

**DOI:** 10.1371/journal.pntd.0007125

**Published:** 2019-02-25

**Authors:** Ana Clara Zoni, Martha Idalí Saboyá-Díaz, Luis Gerardo Castellanos, Rubén Santiago Nicholls, Vendula Blaya-Novakova

**Affiliations:** 1 Independent consultant, Madrid, Spain; 2 Communicable Diseases and Environmental Determinants of Health Department, Pan-American Health Organization/World Health Organization, Washington, DC, United States of the America; University of Connecticut Health Center, UNITED STATES

## Abstract

**Background:**

Yaws is targeted for eradication by 2020 in the WHA66.12 resolution of the World Health Assembly. The objective of this study was to describe the occurrence of yaws in the Americas and to contribute to the compilation of evidence based on published data to undertake the certification of yaws eradication.

**Methodology:**

A systematic review of the epidemiological situation of yaws in the Americas was performed by searching in MEDLINE, Embase, LILACS, SCOPUS, Web of Science, DARE and Cochrane Database of Systematic Reviews. Experts on the topic were consulted, and institutional WHO/PAHO library databases were reviewed.

**Principal findings:**

Seventy-five full-text articles published between 1839 and 2012 met the inclusion criteria. Haiti and Jamaica were the two countries with the highest number of papers (14.7% and 12.0%, respectively). Three-quarters of the studies were conducted before 1970. Thirty-three countries reported yaws case count or prevalence data. The largest foci in the history were described in Brazil and Haiti. The most recent cases reported were recorded in eight countries: Suriname, Guyana, Colombia, Haiti, Martinique, Dominica, Trinidad and Tobago, and Brazil. Gaps in information and heterogeneity were detected in the methodologies used and outcome reporting, making cross-national and chronological comparisons difficult.

**Conclusions:**

The lack of recent yaws publications may reflect, in the best-case scenario, the interruption of yaws transmission. It should be possible to reach the eradication goal in the region of the Americas, but it is necessary to collect more information. We suggest updating the epidemiological status of yaws, especially in two countries that need to assess ongoing transmission. Twenty-four countries need to demonstrate the interruption of transmission and declare its status of yaws endemicity, and sixteen countries should declare if they are yaws-free. It is necessary to formally verify the achievement of this goal in Ecuador.

## Author summary

Yaws is a contagious non-venereal treponematosis caused by the bacterium *Treponema pallidum* subspecies *pertenue*, transmitted by skin contact. It is a poverty-related chronic disease characterized by primary and secondary skin lesions, with latent infection and a chronic stage, which could include a destructive process of bones and joints. Early detection and treatment can avoid gross disfigurement and the associated stigma. Currently, the treatment with a single dose of oral azithromycin has proven effective. This systematic review shows that in the Americas there is a need to update the epidemiological situation of yaws and if needed, to implement and optimize the best public health recommended interventions to interrupt transmission and then verify elimination of transmission. Current WHO guidelines define the methodology for monitoring and evaluating yaws control programs. Yearly surveys of pre-school children, though logistically complex, are needed in the post-zero case surveillance.

## Introduction

Yaws is a poverty-related chronic disease characterized by a primary skin lesion (“mother yaws”) followed by a secondary skin lesion, latent infection, and a chronic stage which may include a destructive process of bones and joints. [1,2]

The disease is a contagious non-venereal treponematosis caused by the bacterium *Treponema pallidum* subspecies *pertenue*, transmitted by skin contact. The incubation period is 9–90 days, with an average of 21 days. Humans are the only source of infection. There is no natural immunity to yaws, and there is no vaccine to prevent it. [1,2] Yaws affects mainly children below 15 years of age (with a peak between 6 and 10 years) and sex differences were not described.[3]

Early detection and treatment can avoid gross disfigurement, which occurs in about 10% of the cases.[1] Nevertheless, yaws remains a cause of disability and associated stigma in much of the developing world, primarily affecting those who reside in tropical regions, in rural and overcrowded communities, living in substandard hygiene conditions, with lack of knowledge of the risk factors for infection, and limited access to healthcare. [4]

Diagnosis should include patient examination and laboratory confirmation with a combination of treponemal and non-treponemal serological tests as the serological tests are indispensable for diagnosing latent disease. It is however also necessary to take into consideration the epidemiological context because the serological tests cannot differentiate between yaws and other treponematoses. [5]

In 1950 the World Health Organization (WHO) estimated that 50 million people were infected with yaws. [6] A review of historical documents from the 1950s shows that over 85 countries and territories were endemic for this disease. The WHO and the United Nations Children’s Fund (UNICEF) provided technical assistance to 46 of these countries between 1952 and 1964, with the consequent drastic decline of yaws prevalence in the endemic areas. [7] Since then, disease control activities were reduced in most countries, and a surveillance phase began, but yaws has not been eradicated.[6,8] Reporting of yaws to the WHO has not been mandatory since 1990 and therefore the availability of up-to-date data on yaws infection is limited.[7] According to WHO, in the Americas, 26 countries were previously considered endemic, and their current status is unknown, seven countries do not have previous history of yaws, and one country–Ecuador–has claimed the interruption of the transmission but it is still necessary to formally verify this achievement. [9,10]

Treatment with a single dose of oral azithromycin has proven effective [11] and has renewed optimism that eradication can be achieved through a new treatment policy, the so-called “Morges Strategy.”[2] This should be implemented along with efforts to facilitate access to clean water, improve sanitation, and promote health education within the community.

Yaws is targeted for eradication, defined as the complete interruption of transmission (absence of new cases of yaws) globally, by 2020 in the WHA66.12 resolution of the World Health Assembly (2013)[12] and by the WHO roadmap on Neglected Tropical Diseases (2012).[13] The Directing Council of the Pan American Health Organization (PAHO) adopted the eradication goal in the CD55.R9 resolution and the plan of action for the elimination of neglected infectious diseases and post-elimination actions 2016–2022.[14,15] WHO details the procedures for verification and certification of interruption of yaws transmission.[3]

To guide the process towards successful eradication, a better knowledge of the historical and current epidemiological status of yaws in the Americas is needed. This review shall allow formulating recommendations and methodological suggestions to move forward on the certification process in the Region.

The objective of this study was to describe the occurrence of yaws in the Americas by age group and by country and to contribute to the compilation of evidence based on published data to undertake the certification of the yaws eradication.

## Methods

A systematic review of the epidemiological situation of yaws in the Americas was performed. An electronic search of the scientific literature published until June 1, 2017, was conducted in the following databases: MEDLINE (PubMed), Embase, LILACS (including SciELO), SCOPUS, Web of Science, Database of Abstracts of Reviews of Effects (DARE) and Cochrane Database of Systematic Reviews. Experts on the topic were consulted, and institutional PAHO/WHO library databases were reviewed.

The search terms used in DARE, Pubmed and EMBASE were “yaws” and “endemic treponematoses”, introduced as MeSH terms or text terms (all fields) or as major terms in EMBASE, together with a combination of the names of all countries, capitals, and main cities of the Region of the Americas introduced as text terms. In LILACS the keywords were also entered in Spanish, Portuguese, and French. Search was limited to studies in humans.

Details of the search strategy are provided in a supplementary file online.

The review was elaborated following the PRISMA (Preferred Reporting Items for Systematic Reviews and Meta-Analyses) Statement criteria for reporting systematic reviews.[16] The review protocol was registered in the PROSPERO database (Reg. N° CRD42017067449) before conducting the study.

The studies included in this review had to fulfill the following criteria: (a) Participants: Persons who have been evaluated clinically or serologically for a diagnosis of yaws. (b) Intervention: Clinical evaluation or serological test for yaws. (c) Outcomes: Number of suspected or confirmed cases (according to the WHO case definition)[2] and/or prevalence of yaws. (d) Study Design: Clinical trials, systematic reviews and meta-analyses, cross-sectional studies, observational studies, and reports of cases. The exclusion criteria were: (1) Studies conducted outside of the Region of the Americas. (2) Studies published in languages other than the official languages of the PAHO Region (English, Spanish, Portuguese, or French). (3) Studies presenting data that had already been included in the review due to their previous publication in another article (duplication).

Prevalence data were only recorded for studies conducted in the community or studies that reported statistical data from epidemiological surveillance.

Two reviewers (ACZ and VBN) carried out the study selection independently, with any disagreements resolved by discussion and consensus. Full-text articles of potentially relevant studies selected through title and abstract screening were analyzed.

For studies that met the inclusion criteria, data were extracted and entered into a Microsoft Excel database. The following information was collected: number of cases (per age group if available), location, year of sample, and setting. For studies not reporting the year in which the survey was carried out, the year of publication was recorded instead.

For studies describing the number of cases or prevalence results by geographical area within several areas of a country or municipality, these data was treated separately rather than as a single data set. Thus, one study may have yielded more than one outcome record.

For studies that did not report results by age group, data were recorded for the total of the population. Results were analyzed separately for children (0–16 years old) and for the general population (includes information from studies that did not report the age of the cases or were conducted in people over 16 years of age).

Mapping was undertaking using Tableau 10.4.

## Results

### Characteristics of the included studies

The initial search identified a total of 679 references. After removing the duplicates, 472 unique references (titles and abstracts) were screened, and 223 papers were selected for full-text reading. Of these, 148 were excluded mainly because they did not report cases. Agreement between the two reviewers was unanimous for the excluded citations. The PRISMA flow diagram of the search strategy is presented in a supplementary file online. Seventy-five full-text articles published between 1839 and 2012 met the inclusion criteria (**Table 1**). Three-quarters of the studies were conducted before 1970.

**Table 1:** Main characteristics of the studies included in the systematic review (n = 75).

**Author** **(publication date)**	Countries or territories	Study design	Setting	Age group	Type of diagnosis	Serological test	Sub-area	Cases (number or range)	Prevalence (%)	Enrollment (year/s or period)	Total population, 1^st^ July 1950 (thousands)*
Rat (1904)[17]	Anguilla	Case series	Health facility	5	1		ND	8		1902	5
Pons (1963)[18]	Argentina	Case report	Health facility	4	3	2	Mendoza; Capital	1		1963	17,150
Jorg (1974)[19]	Argentina	Case report	Health facility	4	4/3	ND	Chaco; Pilcomayo/ Formosa; Mg L.J Fontana	2		1939/ 1960	
Guimaraes (1953)[20]	Brazil	Case series	Health facility	1	3	2	Rio de Janeiro; 2° adm: 5	1,086		1945–1950	53,975
Guimaraes (1961)[21]	Brazil	Cross sectional	Community	5	3	ND	1° adm: 13, 2° adm: 450	515,637	7.1	1956–1959	
Pinotti (1968)[22]	Brazil	Case series	Community	5	ND		Para	18,201	28.4	1956	
Muniz (2012)[23]	Brazil	Cross sectional	Community	5	1		1° adm: 9–11; 2° adm: 78–197	583,515	7.2	1958–1960	
Prussia (1985)[24]	Barbados	Case series	Health facility	4	3	3	ND	1		1982	211
Vargas-Cuellar (1949)[25]	Colombia	Case series	Health facility	1	3	2	Valle Del Cauca	1,281		1938–1939	12,341
Lopez-Narvaez (1956)[26]	Colombia	Cross sectional	Community	6 (0-7/ 8-14/ total)	3	2	Pacific coast and Chocó	111,144		1950–1953	
Hopkins et al. (1977)[27]	Colombia	Case series	Community	5	3	3	Pacific Coast	23,682		1947–1974	
Uribe (1985)[28]	Colombia	Cross sectional	Community	5	1		Pacific Coast; 1° adm: 22	10,747	0.2	1973–1983	
Restrepo et al. (2001)[29]	Colombia	Cross sectional	Community	5	3	3	Pacific Coast; Narino, Cauca, Choco	0		1995	
Pardo-Castello (1939)[30]	Cuba	Cross sectional	Community	6 (0-15/ 16-50/ >50)	3	2	Santiago de Cuba	500	0.0	1937	5,920
Gener (1945)[31]	Cuba	Case series	Health facility	5	3	ND	Sierra Maestra	189		1945	
Moss (1929)[32]	Dominican Republic	Case series	Health facility	5	3	2	Santo Domingo	1,046		1920	2 365
Guderian et al. (1995)[33]	Ecuador	Cross sectional	Community	5	3	3	Esmeraldas; Eloy Alfaro, Santiago Basin; Localities: 87	333	11.2	1988	3,470
Anselmi et al. (1995)[34]	Ecuador	Cross sectional	Community	5	3	3	Esmeraldas; Eloy Alfaro, Santiago Basin; Localities: 87	16	0.6	1993	
Anselmi et al. (2003)[10]	Ecuador	Cross sectional	Community	5	3	3	Esmeraldas; Eloy Alfaro, Santiago Basin; Localities: 87	0	0.0	1998	
Cockin (1913)[35]	Grenada	Case series	Health facility	5	ND		Saint George	360		1912	77
Hartley (1935)[36]	Grenada	Case report	Health facility	3	ND		Saint George	1		1934	
Padilla-Bolaños et al. (1934)[37]	Guatemala	Case report	Health facility	5	4		Suchitepequez; San Pablo Jocopilas	6		1931–1932	3,115
Floch (1945)[38]	French Guyana	Case report	Health facility	4	4		ND and Cayenne	2		1943	25
Sausse (1951)[39]	French Guyana	Case series	ND	5	1		St Lorent du Maroni	17		1951	
Scolnik et al. (2003)[40]	Guyana	Cross sectional	School	6 (1-13/ 15–53)	3	3	Cuyuni/Mazaruni; Mazaruni/Left Bank Essequibo River; Localities: 7	60	4	2000–2001	407
Wilson et al. (1930)[41]	Haiti	Case series	Health facility	6 (0-5/6-10/11-20/≥21)/5	1		Ouest; Port-au-Prince	4,712		1929	3,221
Dwinelle et al. (1947)[42]	Haiti	Case series	Health facility	6 (0-5/6-12/13-16/≥17)	3	2	Ouest; Port-au-Prince and Leogane	500		1944–1945	
Guthe et al. (1951)[43]	Haiti	Case series	Community	5	3	2	Localities: 336	500,000	17.0	1950	
Petrus et al. (1953)[44]	Haiti	Cross sectional	Community	6 (0-16/≥17)	4		Sud and Grand Anse; 2° adm: 5; rural sections/localities: 12	38	0.6	1952	
Hume et al. (1956)[45]	Haiti	Case series	Health facility	1	3	2	Sud-Est; Bainet	1,049		1951–1952	
Samame (1956)[46]	Haiti	Cross sectional	Community	5	4		1° adm: 7; 2° adm: 4	518	0.6	1954–1955	
Petrus et al. (1957)[47]	Haiti	Cross sectional	Community	5	1		1° adm: 7; 2° adm: 5	560	0.5	1952–1955	
Rao (1960)[48]	Haiti	Cross sectional	Community	5	4		1° adm: 5	271	0.3	1958	
La Pommeray et al. (1962)[49]	Haiti	Cross sectional	Community	5	4		1° adm: 4	2	1.1	1960	
PAHO (1962)[50]	Haiti	Cross sectional	Community	5	1/4		National	1,297,868	35.8	1950–56	
							ND	1,368	0.0	1959–62	
Gentilini et al. (1964)[51]	Haiti	Cross sectional	Community	5	ND		National		70.0	1948	
							ND		1.0	1960	
								33		1961	
								0		1963	
Maxwell (1839)[52]	Jamaica	Case report	Health facility	5	1		ND	19		1824–1839	1,403
Turner et al. (1935)[53]	Jamaica	Cross sectional	Community	1	3	2	St Thomas; Bath and Seaforth	2,426	53.5	1932	
Saunders et al. (1936)[54]	Jamaica	Cross sectional	Community/ School	3/ 6 (5–14 yr)	3	2	1° adm: 14; Localities: 48		0–84.0	1932–36	
								12,942	15.1	1936	
Hill (1953)[55]	Jamaica	Case series	Health facility	5	3	2	ND	200		1951–1953	
Ashcroft et al. (1965)[56]	Jamaica	Cross sectional	School	6 (7–15 yr)	3	2	St Mary, Kingston and St Catherine	80	9.2	1963	
Gentle (1965)[57]	Jamaica	Cross sectional	Health facility	1	3	2	1° adm: 12	673		1963	
Gourlay et al. (1965)[58]	Jamaica	Case series	Community	5	3	2	Kingston; Hermitage and August Town (suburban)	4	0.1	1964	
Cummins (1972)[59]	Jamaica	Case series	Health facility	5	3	3	Kingston and Clarendon	20		1972	
Ashcroft et al. (1978)[60]	Jamaica	Cross sectional	School	6 (6–14 yr)	3	3	St Andrews and St Mary		0.6	1976	
Lee et al. (1963)[61]	Saint Lucia	Cross sectional/ Case series	School/ Community	6 (4–15 yr)/ 5	1/ 3	ND	Region 4 and 6	41	3.1	1957	83
							1° adm: 7	3,450		1954–62	
Lee (1967)[62]	Saint Lucia	Cross sectional/ Case series	School/ Community	6 (<15 yr)/ 5	3	ND	Dennery District; Dennery Village and Richfond Valley	294	8.6	1964	
								3		1965–1966	
Montestruc (1953)[63]	Martinique	Case series	Health facility	5	3	2	National	6		1949–1952	222
Maier et al. (2001)[64]	Martinique	Case series	Community	5	2	3	National	13	20.7	1995–1999	
Espinosa et al. (1952)[65]	Panama	Cross sectional	Community	5	ND		Darien; Chepigana and Pinogana; Villages: 16	355	8.7	1949	860
Kooy (1970)[66]	Suriname	Case series	Health facility	5	ND		National	73		1966	215
Niemel et al. (1979)[67]	Suriname	Cross sectional	School	6 (4–16 yr)	3	2	Saramacca	212	21.9	1976	
Niemel et al. (1985)[68]	Suriname	Cross sectional/ Case series	School/ Community/ Health facility	6 (4–16 yr)/ 5	3	3	1° adm: 6	652	7.5	1976–1981	
							National	8,094		1949–1962	
							Paramaribo	71		1973–1981	
Fawkes (1957)[69]	Trinidad and Tobago	Case series	Health facility/ Community	5	3/1	2	1° adm: 15; Localities: 27	11,350		1945–1953	646
							ND	18,825		1888–1920	
								7–13		1924	
								9–11		1932	
Fox (1922)[70]	USA	Case report	Health facility	4	3	2	New York; New York	1		1921	158,804
Alderson et al. (1923)[71]	USA	Case report	Health facility	4	3	2	California; San Francisco	1		1922	
Cady et al. (1924)[72]	USA	Case report	Health facility	4	3	2	Missouri; St. Louis	1		1923	
Downing (1948)[73]	USA	Case report	Health facility	2	3	2	Massachusetts; Suffolk; Boston	2		1947	
Post (1948)[74]	USA	Case report	Health facility	3	3	2	New York; New York	1		1947	
Abreu et al. (1951)	Venezuela	Cross sectional	Community	5	1		Miranda; Paez; Cupira	62	1.2	1949	5,482
							ND	9	0.2	1950	
Medina et al. (1954)	Venezuela	Cross sectional	Community	5	ND		1° adm: 16	18,560	2.4	1943–1950	
Smith et al. (1971)[75]	Venezuela	Case series	Health facility	5	3	3	Distrito Federal; Libertador (Caracas)	71		1969	
PAHO (1933)[76]	5	Case series	Health facility /ND	6 (0–13)/5	ND		1° adm: 5	3–61,931		1918–1933	
PAHO (1935)[77]	Colombia/ Jamaica/ Panama	Case series	ND/ Health facility / Community	5	ND		Choco	4,000		1930–1934	
							National/ Subnational, Localities: 5	9–61	0.6–16.4	1935	
PAHO (1936)[78]	5	Case series	ND	6 (0–15)/5	ND		National and subnational	1–651		1936	
PAHO (1937)[79]	7	Case series	School/ ND	6 (children)/5	ND		National and subnational	0–95,779		1937	
PAHO (1939)[80]	3	Case series	ND	6(0-5/5-10/10-20)/5	3/ND	ND	National and subnational	260–4,690		1939	
Samame (1952)[81]	15	Case series	ND	5	ND		National and subnational		0–4.9	1952	
Bica et al. (1957)[82]	40	Case series	ND	5	ND		National	0–528,243		1937–1956	
Hopkins (1977)[83]	5	Case series	ND	5	ND		National and subnational	4–61,353		1950–1975	
WHO (1981)[84]	18	Case series	ND	5	ND		National	0–4,742		1971–1979	
WHO (1984)[85]	13	Case series	ND	5	ND		National	1–627		1977–1982	
Antal et al. (1985)[6]	15	Case series	ND	5	ND		National	0–2,065		1971–1981	
St John (1985)[86]	Saint Vincent and the Grenadines	Case series	ND	5	ND		National and subnational	18	0.0	1956–1958	67
	Suriname/Colombia							120–215		1979–1983	
Meheus et al (1992)[8]	7	Case series	Community/ School/ Health facility	6 (school-children)/5	ND		National	0–241		1977–1992	

**ND:** Not documented

**Subarea:** Name of the 1^st^ administrative level; number of municipalities or 2^nd^ administrative level; number of localities (cities or villages).

**Age groups:** (1) WHO age range categories recommended (0–4 /5-9/ 10–14 /≥15 years); (2) 5–9 years exclusively; (3) 10–14 years exclusively; (4) ≥ 15 years exclusively; (5) when studies did not report age group differences; (6) other age group classification.

**Type of diagnosis:** (1) Clinical diagnosis exclusively; (2) Serological diagnosis exclusively; (3) Clinical and serological diagnosis, with or without darkfield/histology examination; (4) Clinical and darkfield/ histology examination.

**Serological test:** (1) treponemal test; (2) non-treponemal; (3) treponemal test +non treponemal test.

(*) United Nations, Department of Economic and Social Affairs, Population Division (2017). World Population Prospects: The 2017 Revision, DVD Edition. Available at: https://population.un.org/wpp/Download/Standard/Population/

**Fig 1:**
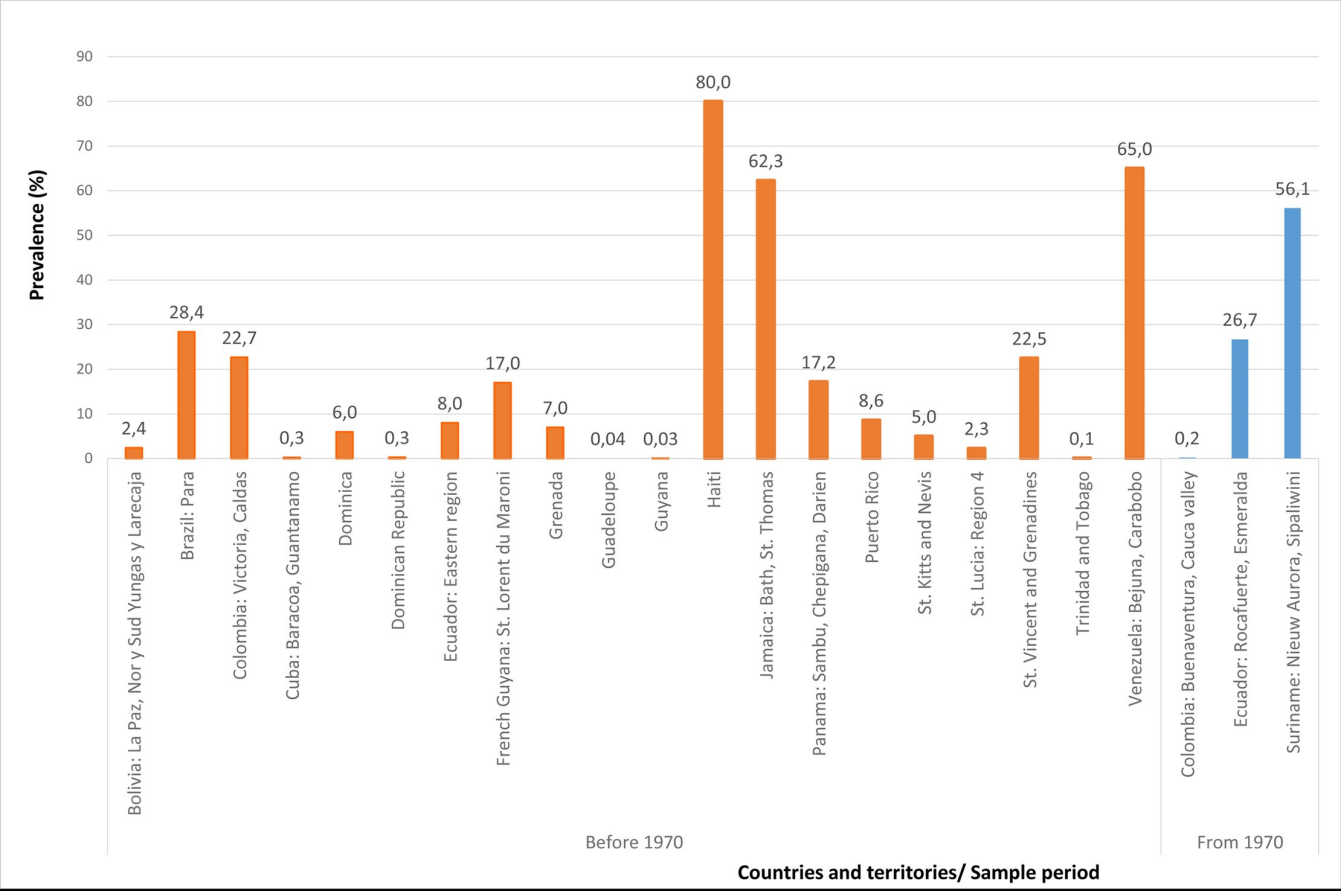
Prevalence of yaws (maximum) in general population by country or territory and sample period.

Epidemiological data were found for 43 countries **(Table 2 and Fig 1)**. The scientific literature mainly stemmed from Haiti (n = 11, 14.7%) and Jamaica (n = 9, 12.0%). Thirteen studies (17.3%) reported cases from various countries.

**Table 2:** Number of cases of yaws per year (range) in general population by country or territory and sample period.

Subregion	Country or territory	Sample periods	
		Before 1970	After 1970
**North America**	Canada	0	
	United States of America	0–3	
**Central American Isthmus**	Belize	0	
	Costa Rica	6–40	
	El Salvador	0–1	
	Guatemala	1–6	0–1
	Honduras	0	
	Nicaragua	0–4	
	Panama	1–104	0–1
**Latin Caribbean**	Cuba	189–4,000	
	Dominican Republic	1,046–3,827	0–9
	French Guiana	0–17	
	Guadeloupe	100–501	
	Haiti	0–1,281,666	11–123
	Martinique	6	2–40
	Puerto Rico	0–94	
Andean Area* **and Brazil**	Colombia	8,622–68,725	1–573
	Ecuador	541–13,651	0–868
	Peru	555–2,797	1–23
	Venezuela	9–10,235	0
	Brazil	2–297,681	2–2,996
**Southern Cone**	Argentina	0–3	
	Chile	0	
	Paraguay	0	
	Uruguay	0	
Non-Latin Caribbean*	Anguilla	8	
	Antigua and Barbuda	0–70	0–9
	Aruba	0	
	Bahamas	0	
	Barbados	0	1
	Curacao	0	
	Dominica	1,031–1,469	3–351
	Grenada	360–1,500	0–15
	Guyana	25–72	0–36
	Jamaica	4–8,500	0–62
	Montserrat	0	
	Saint Lucia	0–1,124	0–26
	Saint Vincent and the Grenadines	5–1,117	0–96
	Suriname	73–1,436	0–45
	Trinidad and Tobago	16–8,069	0–1,048
	Virgin Islands (UK)	1	

General Population includes information from studies that did not report the age of the cases or were conducted in adult population only.

*Bolivia and Saint Kitts and Nevis only reported prevalence data before 1970

More than half of the studies were case series or case reports (n = 46, 61.3%) followed by cross-sectional design (n = 26, 34.7%), and three studies (4.0%) had a mixed design (both case-series and cross-sectional design).

More than half of the studies did not report the age of the cases (n = 42, 56.0%) or were conducted in adult population only (n = 7, 9.3%). Twenty-six studies reported cases in children up to 16 years old (34.7%). Categorization into four categories according to age as recommended by the WHO (0–4; 5–9; 10–14; ≥15 years old) was performed in five articles (6.8%). The remaining 21 studies that reported cases in children (28.0%) either used other age categories or did not disaggregate the results into age groups.

There was a notable heterogeneity in the diagnostic method used: 48.0% of the studies (n = 36) performed both clinical and serological diagnosis in combination with or without dark-field or histology examination; 10.7% of the studies (n = 8) used solely clinical diagnosis; 8.0% of the studies (n = 6) used clinical diagnosis with dark-field or histology examination; one study in children used serological diagnosis exclusively (1.3%); and five studies reported cases identified through more than one diagnostic algorithm (6.7%). The diagnostic methods were not documented in 19 studies (25.3%).

From the 41 studies that mentioned having used a serological test, most used non-treponemal tests (n = 23, 56.1%) or combined non-treponemal/treponemal tests (n = 12, 29.3%), and six articles (14.6%) did not specify the kind of serological test used.

### Countries with reports of yaws data 1839–2012

According to the filiation of countries to the PAHO, there are 35 Member States, four Associate Members, and three Participant States with 12 territories in the Region of the Americas. [87] For this study we will call them countries and territories. Information was identified for 43 countries and territories of the Americas (**Table 2 and Fig 1**). Yaws case counts were reported in 31 countries: one from North America, five from Central America, seven from Latin Caribbean, five from Andean Area and Brazil, one from Southern Cone, and twelve from the Non-Latin Caribbean. Yaws prevalence data were reported in 20 countries which included Bolivia and Saint Kitts and Nevis.

The absence of yaws was reported from 10 countries or territories: Aruba, Bahamas, Belize, Canada, Chile, Curacao, Honduras, Montserrat, Paraguay, and Uruguay. No data were available for seven countries or territories: Bermuda, Turks and Caicos, Bonaire, Saba, Saint Eustatius, Sint Maarten, and the Cayman Islands. Although information for Mexico was not found in this review, according to the information from the PAHO no cases of yaws have ever been reported in Mexico. [82,83]

### Report of yaws in general population

The reported number of cases of yaws and the highest prevalence of yaws in general population by country or territory and sample period are summarized in **Table 2** and **Fig 1,** respectively. Taking into account that three-quarters of the studies were conducted before 1970, the information was summarized into two groups according to the period (before 1970 and since 1970 to date).

General Population includes information from studies that did not report the age of the cases or were conducted in adult population only.

Before 1970 (32 countries or territories described at least one case in this period):

In the **United States of America,** three cases of yaws were reported between 1921 and 1923. In **Costa Rica,** a total of 40 cases were described in the province of Puntarenas in 1929, and six cases were detected at the eastern border of the country between 1951 and 1956. In **El Salvador,** one case was reported in 1936. **Guatemala** reported a maximum of six cases in San Pablo Jocopilas in 1931. **Nicaragua** reported four cases in 1936. In **Panama,** the highest case number was 104 cases (prevalence 17.2%) in Sambu in Chepigana, Darien, in 1949. In **Cuba,** an estimated maximum of 4,000 cases was recorded in 1953 (last data available), principally in the Eastern Province. The **Dominican Republic** reported a maximum of 3,827 cases in 1955–1956. Seventeen cases of yaws were observed in the villages of the Maroni Basin tropical rainforest in **French Guiana** in 1951, and the last national report from 1954 included eight cases. **Guadeloupe** had a historic maximum of 501 cases in 1932, and the last report from 1950–1953 informed of 100 cases (0.04% of the total population). **Haiti** reported the highest nation-wide prevalence of yaws (80.0%) in 1948 and a maximum of 1,281,666 cases in the period 1950–1954. **Martinique** reported six cases between 1949 and 1952. **Puerto Rico** reported a maximum of 94 cases in 1936; the last report from 1956 reported no new cases. A yaws prevalence of 2.4% in the general population was recorded in the La Paz department in **Bolivia** in 1946. **Colombia** reported a maximum of 68,725 cases between 1950 and 1953 on the Pacific Coast and in Chocó. **Ecuador** reported a maximum of 13,651 cases in the Coastal Region between 1950 and 1955. In **Peru,** a maximum of 2,797 cases was reported from the eastern part of the country in the period 1952–1954. **Venezuela** reported a maximum of 10,235 cases from 1951 to 1955, primarily from the States of Miranda, Sucre, Yaracuy, Cojedes, and Carabobo. **Brazil** reported a maximum of 297,681 cases from 12 states (197 municipalities) in 1957. Most of these cases were concentrated in the Northeastern Region. The highest prevalence rate reached 28.4% in Para (1956–1959). In **Argentina,** three cases were reported between 1939 and 1963. **Anguilla** reported eight cases in 1902. **Antigua and Barbuda** reported a historic maximum of 70 cases in 1954. **Dominica** reported a historic maximum of 1,469 cases in 1954. **Grenada** reported a maximum of 1,500 cases (7.0% of the total population) from 1950 to 1953. **Guyana** reported a prevalence of yaws of 0.03% in 1952 and 72 cases in 1954. **Jamaica** reported a maximum of 8,500 cases in 1955 and the highest prevalence (62.3%) was described in St. Thomas, Bath, in 1932. **Saint Kitts and Nevis** reported a national yaws prevalence of 5% in a total population of 55,000 in 1956. **Saint Lucia** reported a historic maximum of 1,124 cases in 1954. **Saint Vincent and the Grenadines** reported a maximum of 1,117 cases (22.5% of the population) in 1956. In **Suriname,** a historic maximum of 1,436 cases was recorded in 1950. **Trinidad and Tobago** reported a total of 8,069 cases from 1946 to 1953 (with a prevalence of 0.1% in 1952). For the **Virgin Islands (UK),** sporadic cases of yaws were described in 1956.

Since 1970 (19 countries or territories described at least one case in this period): **Guatemala** reported one case in 1975, and the last update from 1979 reported no cases. **Panama** reported one case in 1977, and the last update from 1980 reported no cases. The **Dominican Republic** reported nine cases in 1971, and the last update from 1979 reported no cases. In **Haiti,** the case count decreased from 123 in 1971 to 31 in 1979 (last data available). In **Martinique,** a peak of 40 cases was recorded in 1975, and the last report from 1995–1999 cited 13 cases detected in blood donors. **Colombia** reported 573 cases on the Pacific Coast in 1973 and a prevalence of 0.2% in Buenaventura, Cauca Valley, in 1981. In 1992, 108 cases were reported, and the last update from 1995 reported no cases. **Ecuador** reported a maximum of 868 cases in 1975. Three epidemiological surveys conducted in the same 87 communities from the Santiago Basin Esmeraldas revealed the highest percentage of people affected by yaws in Rocafuerte (26.7%) in 1988 and all the communities reported zero cases in 1998. The last two cases in **Peru** were diagnosed in 1979. In **Brazil,** the number of cases described ranged from two in the state of Rondõnia (1972) to 2,996 in Amazonas (1970). The last report from 1977 described 17 cases. **Antigua and Barbuda** reported nine cases in 1972, and the last case was reported in 1977. One case was reported in **Barbados** in 1982. **Dominica** reported 351 cases in 1971 and 28 cases in 1979 (last report identified). **Grenada** reported 15 cases in 1971 and the last report from 1979 identified no cases. **Guyana** reported 36 cases in the period 1979–1984. The last report from 2002 from Mazaruni/Left Bank Essequibo River did not report any new cases. **Jamaica** reported 62 cases in 1972, and the last report with no new cases dates from 1979. **Saint Lucia** reported 26 cases in 1971 and the last case was reported in 1979. **Saint Vincent and the Grenadines** reported 96 cases in 1972 and the last case was reported in 1979. In **Suriname,** 45 and 21 cases were reported in 1982 and 1983, respectively (last data available). **Trinidad and Tobago** reported a maximum of 1,048 cases in 1974 and the last report identified included 123 cases in 1979.

### Report of yaws in children

Given the heterogeneity of the age group reporting in children, results were analysed without disaggregating into subgroups of age.

Yaws infection in children was described in 12 of the 43 countries or territories in which data were recorded (**Table 3**). In the entire study period, the highest case count reported in the community setting was of 42,419 children on the Pacific Coast and in Chocó, Colombia (1950–1953). The highest prevalence (90%) was found in Brandon Hill, Saint James, Jamaica, in 1932.

**Table 3:** Number of cases and prevalence of yaws (range) in children (0–16 years old) by country or territory, setting and sample period.

Country or territory	Setting	Sample periods			
		Before 1970		After 1970	
		Cases (n)	Prevalence (%)	Cases (n)	Prevalence (%)
Brazil	Health facility	521			
	ND	272			
Colombia	Community	42,419			
	School	824	70.1		
	Health facility	579			
Cuba	Community	224			
Grenada	Health facility	1			
Guyana	School			8–52	1.5–5.1
Haiti	Community	0–718	0–2.6		
	Health facility	114–1,053*			
Jamaica	Community	228–496	0–90.0		
	School	24–2,354	0.15–38.6	2–3	0.29–1.5
	Health facility	369			
Panama	Health facility	3			
Saint Lucia	School	9–237	1.3–13.2		
Suriname	School			2–398	0.2–42.1
United States of America	Health facility	3			
Venezuela	ND	77*			

* Includes children from 0 to 20 years old; ND: Not documented

In the school setting, the highest case number recorded was 2,354 cases from 64 schools in Saint Catherine, Jamaica, in 1932. The highest prevalence rate reached 70.1% (824 children) reported from 29 schools in Cauca, Colombia, in 1937.

The highest number of cases identified in a health facility setting was reported from Port-au-Prince, Haiti, in 1929 (1,053 children).

## Discussion

This systematic review compiles epidemiological information (occurrence of yaws) for 43 countries and territories of the Americas. Included studies originated primarily from two countries in the Caribbean (Haiti and Jamaica) and were published between 1839 and 2012.

Data show that before 1970, the yaws infection was well established in many countries or territories of the Americas, with cases described in 32 countries. Several large foci of the disease were observed in the first half of the 20^th^ century. For example, 61,931 cases were reported in Colombia (1918–1927)[76] and 25,186 cases in Brazil (Teofilo Otoni, Minas Gerais; 1934–1941).[20] The efforts of some of the countries such as Colombia, Suriname or Jamaica to control yaws with arsenical or bismuth treatment in the pre-antibiotic era appear not to have had a large impact on public health as the prevalence of the yaws infection remained high in the subsequent decades in these countries.

In the fifties and sixties, yaws infection continued to afflict the Region. The largest foci described in the history were located in Brazil and Haiti, with both countries reporting more than 500,000 cases.[23,50] The large number of studies and cases identified for these two decades probably reflects the greater political commitment fostered internationally by the WHO and UNICEF [7] and increased provision of economic and human resources for surveying extensive areas, undertaking population treatment campaigns, establishing surveillance systems, and publishing research papers and evaluation reports, rather than an actual spread of the disease.

The information encountered suggests that the yaws spread got under control during the following two decades on this continent: the number of countries or territories with yaws cases decreased down to 19, with less than 1,000 cases reported from each one of them except for Brazil [83] and Trinidad and Tobago.[84] This decline seems to be related to the success of the above-mentioned mass penicillin treatment campaigns in the 1950s.[8] In the 1970s, the yaws programs were integrated into primary health care systems in some countries, but in many others, they became weak or almost non-existent. [7]

Since 1990, when mandatory reporting of yaws cases to the WHO was rescinded, the data have been scarce: information has been available for six countries only, and low-level transmission of yaws was reported from Colombia,[8] Martinique,[64] and Guyana.[40] In Venezuela and Panama, no yaws cases have been identified since 1977 and 1980, respectively.[8] After two well-conducted epidemiological surveys in 1993 and 1998 that reported an absence of clinical and serological cases, Ecuador may be considered a yaws-free country, but formal WHO verification of the interruption of transmission is needed.[10] Yaws information from other countries has become outdated. [7] The last update on the status of endemicity of yaws of the Global Health Observatory data repository of the WHO did not report cases from any country of the Americas for the period 2008 to 2013.[9]

According to the information provided by the PAHO’s Regional Neglected Infectious Diseases Program, from 21 countries that responded to a survey in 2017 (initiative of the WHO to compile information globally for yaws eradication purposes), only Bermuda, Jamaica, and Nicaragua have included yaws in their current surveillance system. No country reported having national guidelines or a policy document for yaws except Nicaragua, which has included yaws in its notifiable diseases reporting system. No country in the Region of the Americas has identified any yaws cases between 2014 and 2017 except for Colombia and Haiti. Colombia identified less than 100 yaws cases (without diagnostic confirmation) based on data retrieved from the health information system using the yaws codes of the 10th revision of the International Classification of Diseases (ICD-10); the monitoring of the events and the anti-yaws campaign were dismantled in 2001. Haiti reported seven cases detected in the Department of Grand’Anse in 2015.

The reduction of the number of countries and territories with reports of cases or prevalence of yaws published before 1970 (n = 32) and after 1970 (n = 19) by almost 60% is remarkable. The lack of recent yaws publications may reflect, in the worst-case scenario, a) that the countries face difficulties in publishing yaws data in indexed journals; b) a lack of capacities, interest or funding to conduct epidemiological surveys; and/ or c) a lack of political interest for undertaking further surveys as the issue of yaws is not considered a priority due to more pressing public health problems or the increasing relevance of other infectious diseases (e.g., chikungunya or zika). In the best-case scenario, it may be indicative of the interruption of the yaws transmission which could be related to the impact of the disease eradication interventions and improvements in the socioeconomic status in many countries. However, in many countries, the epidemiological surveillance, which is key to detecting and responding to a possible resurgence of the disease and to certifying the interruption of the transmission, stopped after 1970. The criterion proposed by the WHO for confirming that transmission of yaws in an area has ceased is the absence of new cases for a continuous period of three years of clinical and serological (in children under five years of age) surveillance. [2]

Given that this is a review without date limits and with flexibility in the type of studies included, we detected gaps in information and considerable heterogeneity in the methodologies used and outcome reporting. Thus, the comparison between countries must be carried out with caution: (a) In spite of the fact that cases were registered by the smallest geographic unit identified whenever possible, information was frequently found at the national level only. (b) In many studies, the prevalence reported had to be calculated over the total number of persons examined instead of over the total population ascertained by a census. (c) Several studies, especially those that included data reported to the WHO by national health authorities, did not specify whether the cases reported were active only or also latent,[84] when it is known that the ratio of clinical to latent cases is around 1:6.[88] Moreover, half of the studies either did not use serology in the diagnostic protocol or did not mention its use, not allowing distinguishing between suspected or confirmed cases, yet only surveys with clinical and serological diagnosis can provide data on the actual level of endemicity in a population.[4] Therefore, the information identified in this review can mainly indicate the mere existence of cases or foci of yaws in a determined country in a specified period. [84]

There was a large variation in the age of the cases reported and in the age range categorization. More than half of the studies did not report the age of the cases, 26 studies reported cases in children, and only five studies grouped participants by age into four categories as recommended by the WHO.[2] The separate analysis of children and adult data is essential because on one hand, it is known that children under 15 years of age are the main reservoir of the infection;[88] and on the other hand, it detects the occurrence of new infections and avoids the complexity of dealing with adult population that was successfully treated but remains seropositive. [4] Yearly surveys of pre-school children, though logistically complex, are needed in the post-zero case surveillance.

**Fig 2:**
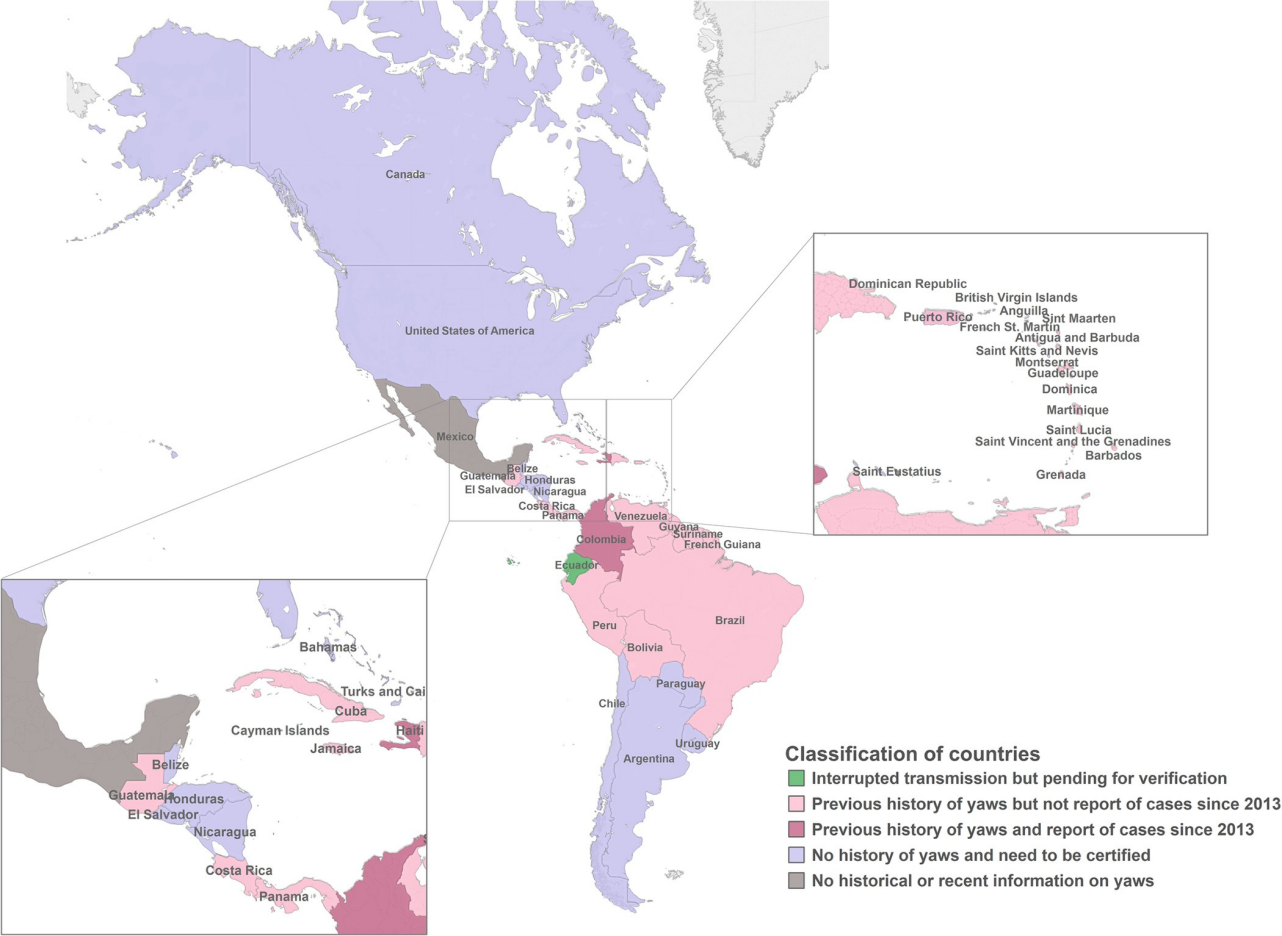
Classification of countries for certification of interruption of yaws transmission in the Americas.

Based on this review, and by combining the results to the classification of countries for verification of interruption of yaws transmission proposed by WHO [3], we suggest the following actions be carried out in the countries of the Americas (Fig 2):

### Countries that have interrupted transmission and need to be verified

Ecuador has claimed the achievement of the goal. It would be recommendable to reassess the interruption of transmission in children aged 1–5 years in areas were yaws was historically endemic. The epidemiological and historical information supporting the achievement of the interruption of transmission of yaws in the country should be compiled in a dossier and submitted to the WHO for the verification process. Ecuador may be the first country in the Americas to apply the PAHO/WHO procedures for verification and certification of interruption of transmission of yaws.

### Countries with no history of autochthhonous infectious cases of yaws that need to be certified

Six countries reported less than ten imported cases before 1970 (Argentina, 1963; Nicaragua, 1957; Virgin Island (UK) 1956; the United States of America, 1947; El Salvador, 1936; and Anguilla, 1902). Also, ten countries reported zero cases before 1970 (Canada, Belize, Honduras, Chile, Paraguay, Uruguay, Aruba, Bahamas, Curacao, and Monserrat). We did not find reports of yaws cases after 1970 in all these countries. Documentation to support that autochthhonous cases of yaws have ever occurred should be compiled and submitted to PAHO/WHO in all these countries along with the evidence that its health and surveillance systems are sufficient to detect any imported yaws case.

### Countries with history of yaws but no report of cases since 2013

Cuba, Guadeloupe, Puerto Rico, Costa Rica and French Guiana reported cases, and Bolivia and Saint Kitts and Nevis reported prevalence before 1970. Peru, Antigua and Barbuda, Barbados, Saint Lucia, and Saint Vincent and the Grenadines have reports of one or two cases of yaws after 1970, most of them before 1980. Guyana, Suriname, Dominica, Trinidad and Tobago, Brazil and Martinique reported cases after 1970, most of them before 1990. Guatemala, Panama, Dominican Republic, Grenada, Jamaica, and Venezuela have reports of zero cases in studies carried out after 1978. These countries should review in depth the information at local level, mainly in the municipalities with a history of yaws. These countries should provide comprehensive evidence to support the elimination in a dossier, and declare the status of yaws endemicity indicating whether there is evidence of the interruption of transmission or whether additional assessment needs to be carried out.

### Countries with history of yaws and report of cases since 2013

Although Colombia had the latest publication of yaws in 1992, the country reported suspected cases between 2014 and 2017 to PAHO/WHO. Haiti also reported cases in 2015 to PAHO/WHO. In these countries, it would be necessary to carry out an assessment including the collection of information to support the absence of the disease, review past and existing records, and conduct clinical and serological surveys in children in previously endemic areas. Countries with confirmed cases should implement the Morges Strategy.

### Countries with no historical or recent information on yaws

For seven countries there was no data available on the occurrence of yaws: Bermuda, Turks and Caicos, Bonaire, Saba, Saint Eustatius, Sint Maarten, and the Cayman Islands. A more in-depth search of information, studies or data published locally should be carried out in each one to decide on next steps. Although Mexico reported no cases of yaws since 2013 to PAHO/WHO, the country should compile all the information to support its current epidemiological status.

The proposed classification of countries is intended to encourage countries in the Americas to start reviewing and documenting the current epidemiological situation of yaws. In conclusion, it should be possible to reach the eradication goal in the region of the Americas, but it is necessary to update the epidemiological status and evidence must be compiled to confirm whether the interruption of yaws transmission has occurred in most of the countries.

## Supporting information

S1 Table Search strategy. (DOCX)

S2 Table PRISMA checklist. (DOCX)

S1 Fig PRISMA flow diagram. (DOCX)
