# Supplementary material for: Epidemiological situation of yaws in the Americas: A systematic review in the context of a regional elimination goal
Source: PLoS Negl Trop Dis. 2019 Feb 25;13(2):e0007125. doi: 10.1371/journal.pntd.0007125 (PMC6405159; doi:10.1371/journal.pntd.0007125)
Supplement: S1 Table — (DOCX) [file pntd.0007125.s001.docx]

**Search Strategy**

| **Data sources** | **Question (Keywords)** |
| --- | --- |
| PUBMED | (((yaws[MeSH Terms]) OR "endemic treponematoses")) AND (("Latin America*" OR "South America" OR "Central America" OR caribbean OR "North America" OR anguilla OR "Antigua and Barbuda" OR argentina OR aruba OR bahamas OR barbados OR belize OR bermuda OR Bolivia* OR Brazil* OR "British Virgin Islands" OR "Cayman Islands" OR Chile* OR Colombia* OR "Costa Rica" OR Cuba* OR dominica OR "Dominican Republic" OR "El Salvador" OR ecuador OR "French Guiana" OR grenada OR guadalupe OR guatemala OR guyana OR haiti OR honduras OR Jamaica* OR martinique OR mexico OR montserrat OR "Netherlands Antilles" OR Nicaragua* OR panama OR Paraguay* OR peru OR "Puerto Rico" OR "Saint Kitts and Nevis" OR "Saint Lucia" OR "Saint Vincent and the Grenadines" OR suriname OR "Trinidad and Tobago" OR "Turks and Caicos Islands" OR Uruguay* OR venezuela OR brasil* OR argentinean OR mexican OR costaric* OR "the Valley" OR "Saint John's" OR "Buenos Aires" OR basseterre OR basse-terre OR hamilton OR oranjestad OR nassau OR bridgetown OR belmopan OR sucre OR "La Paz" OR brasilia OR "Sao Paulo" OR "Rio de Janeiro" OR "Belo Horizonte" OR maceio OR manaus OR goiania OR belem OR "Porto Alegre" OR florianopolis OR "George Town" OR santiago OR valparaiso OR concepcion OR temuco OR bogota OR cali OR medellin OR barranquilla OR "San Jose" OR havana OR habana OR "Santo Domingo" OR roseau OR "San Salvador" OR quito OR guayaquil OR "Saint George's" OR "Port au Prince" OR "Port of Spain" OR tegucigalpa OR kingston OR kingstown OR willemstad OR managua OR asuncion OR lima OR "San Juan" OR marigo OR castres OR paramaribo OR montevideo OR caracas OR "Road Town" OR mexicano OR "Tuxtla Gutierrez" OR chihuahua OR guanajuato OR guadalajara OR toluca OR morelia OR monterrey OR puebla OR "United States" OR california OR sacramento OR colorado OR denver OR connecticut OR hartford OR delaware OR dover OR florida OR tallahassee OR georgia OR atlanta OR hawaii OR honolulu OR idaho OR boise OR illinois OR springfield OR indiana OR indianapolis OR iowa OR "Des Moines" OR kansas OR topeka OR kentucky OR frankfort OR louisiana OR "Baton Rouge" OR maine OR augusta OR maryland OR annapolis OR massachusetts OR boston OR michigan OR lansing OR minnesota OR "Saint Paul" OR mississippi OR jackson OR missouri OR "Jefferson City" OR montana OR helena OR nebraska OR lincoln OR nevada OR "Carson City" OR "New Hampshire" OR concord OR "New Jersey" OR trenton OR "New Mexico" OR "Santa Fe" OR "New York" OR albany OR "North Carolina" OR raleigh OR "North Dakota" OR bismarck OR ohio OR columbus OR oklahoma OR "Oklahoma City" OR oregon OR salem OR pennsylvania OR harrisburg OR "Rhode Island" OR providence OR "South Carolina" OR columbia OR "South Dakota" OR pierre OR tennessee OR nashville OR texas OR austin OR utah OR "Salt Lake City" OR vermont OR montpellier OR virginia OR richmond OR washington OR olympia OR "West Virginia" OR charleston OR wisconsin OR madison OR wyoming OR cheyenne OR canada OR ontario OR quebec OR "British Columbia" OR alberta OR manitoba OR saskatchewan OR "Nova Scotia" OR "New Brunswick" OR "Newfoundland and Labrador" OR "Prince Edward Island" OR "Northwest Territories" OR nunavut OR yukon OR ottawa OR toronto OR "Quebec city" OR halifax OR fredericton OR winnipeg OR victoria OR charlottetown OR regina OR edmonton OR "St. John's" OR yellowknife OR whitehorse OR iqaluit)) **Filters:** Humans |
| EMBASE | 'endemic treponematoses' OR 'yaws'/exp/mj AND 'south and central america'/exp OR 'south and central america' OR 'south america'/exp OR 'south america' OR 'central america'/exp OR 'central america' OR 'latin america'/exp OR 'latin america' OR 'latin american' OR 'caribbean islands'/exp OR 'caribbean islands' OR 'caribbean'/exp OR 'caribbean' OR 'african caribbean'/exp OR 'african caribbean' OR 'anguilla (country)'/exp OR 'anguilla (country)' OR 'antigua and barbuda'/exp OR 'antigua and barbuda' OR puebla OR monterrey OR morelia OR toluca OR 'guadeloupe'/exp OR 'guadeloupe' OR guadalajara OR guanajuato OR chihuahua OR 'tuxtla gutierrez' OR mexicali OR 'road town' OR caracas OR montevideo OR paramaribo OR castries OR marigot OR 'san juan' OR lima OR 'aruba'/exp OR 'aruba' OR 'argentina'/exp OR 'argentina' OR asuncion OR managua OR willemstad OR kingstown OR kingston OR tegucigalpa OR 'port of spain' OR 'port au prince' OR 'saint georges' OR guayaquil OR quito OR 'san salvador' OR roseau OR 'santo domingo' OR habana OR havana OR 'san jose' OR barranquilla OR medellin OR cali OR bogota OR temuco OR concepcion OR valparaiso OR santiago OR 'george town' OR florianopolis OR 'porto alegre' OR belem OR goiania OR manaus OR maceio OR 'belo horizonte' OR 'rio de janeiro' OR 'sao paulo' OR brasilia OR 'la paz' OR sucre OR belompan OR bridgetown OR nassau OR oranjestad OR hamilton OR 'basse terre' OR basseterre OR 'buenos aires' OR 'saint johns' OR 'the valley' OR costaric* OR 'mexican' OR argentinean OR brasil* OR 'venezuela'/exp OR 'venezuela' OR uruguay* OR 'uruguay'/exp OR 'uruguay' OR 'turks and caicos islands'/exp OR 'turks and caicos islands' OR 'trinidad and tobago'/exp OR 'trinidad and tobago' OR 'suriname'/exp OR 'suriname' OR 'saint vincent and the grenadines'/exp OR 'saint vincent and the grenadines' OR 'saint lucia'/exp OR 'saint lucia' OR 'saint kitts and nevis'/exp OR 'saint kitts and nevis' OR 'puerto rico'/exp OR 'puerto rico' OR 'peru'/exp OR 'peru' OR paraguay* OR 'paraguay'/exp OR 'paraguay' OR 'panama'/exp OR 'panama' OR nicaragua* OR 'nicaragua'/exp OR 'nicaragua' OR 'netherlands antilles'/exp OR 'netherlands antilles' OR 'montserrat'/exp OR 'montserrat' OR 'mexico'/exp OR 'mexico' OR 'martinique'/exp OR 'martinique' OR jamaica* OR 'jamaica'/exp OR 'jamaica' OR 'honduras'/exp OR 'honduras' OR 'haiti'/exp OR 'haiti' OR 'guyana'/exp OR 'guyana' OR 'guatemala'/exp OR 'guatemala' OR guadalupe OR 'grenada'/exp OR 'grenada' OR 'french guiana'/exp OR 'french guiana' OR 'ecuador'/exp OR ecuador OR 'el salvador'/exp OR 'el salvador' OR 'dominican republic'/exp OR 'dominican republic' OR 'dominica'/exp OR 'dominica' OR cuba* OR 'cuba'/exp OR 'cuba' OR 'costa rica'/exp OR 'costa rica' OR colombia* OR 'colombia'/exp OR 'colombia' OR chile* OR 'chile'/exp OR 'chile' OR 'cayman islands'/exp OR 'cayman islands' OR 'british virgin islands'/exp OR 'british virgin islands' OR brazil* OR 'brazil'/exp OR 'brazil' OR bolivia* OR 'bolivia'/exp OR 'bolivia' OR 'bermuda'/exp OR 'bermuda' OR 'belize'/exp OR 'belize' OR 'barbados'/exp OR 'barbados' OR 'bahamas'/exp OR 'bahamas' OR 'united states'/exp OR 'united states' OR california OR sacramento OR colorado OR denver OR connecticut OR hartford OR delaware OR dover OR florida OR tallahassee OR georgia OR atlanta OR hawaii OR honolulu OR idaho OR boise OR illinois OR springfield OR indiana OR indianapolis OR iowa OR 'des moines' OR kansas OR topeka OR kentucky OR frankfort OR louisiana OR 'baton rouge' OR maine OR augusta OR maryland OR annapolis OR massachusetts OR boston OR michigan OR lansing OR minnesota OR 'saint paul' OR mississippi OR jackson OR missouri OR 'jefferson city' OR montana OR helena OR nebraska OR lincoln OR nevada OR 'carson city' OR 'new hampshire' OR concord OR 'new jersey' OR trenton OR 'new mexico' OR 'santa fe' OR 'new york' OR albany OR 'north carolina' OR raleigh OR 'north dakota' OR bismarck OR ohio OR columbus OR oklahoma OR 'oklahoma city' OR oregon OR salem OR pennsylvania OR harrisburg OR 'rhode island' OR providence OR 'south carolina' OR columbia OR 'south dakota' OR pierre OR tennessee OR nashville OR texas OR austin OR utah OR 'salt lake city' OR vermont OR montpellier OR virginia OR richmond OR washington OR olympia OR 'west virginia' OR charleston OR wisconsin OR madison OR wyoming OR cheyenne OR 'canada'/exp OR canada OR quebec OR 'british columbia' OR alberta OR manitoba OR saskatchewan OR 'nova scotia' OR 'new brunswick' OR 'newfoundland and labrador' OR 'prince edward island' OR 'northwest territories' OR nunavut OR yukon OR ottawa OR toronto OR 'quebec city' OR halifax OR fredericton OR winnipeg OR victoria OR charlottetown OR regina OR edmonton OR 'st johns' OR yellowknife OR whitehorse OR iqaluit OR 'north america'/exp AND 'human'/de |
| LILACS | tw:((mh:(buba)) OR (mj:(pian)) OR (tw:("treponematosis endemicas"))) AND (instance:"regional") AND ( limit:("humans"))  w:((mh:(buba)) OR (mj:(pian)) OR (tw:("treponematosis endemicas"))) AND (instance:"regional") AND ( limit:("humans") AND pais_assunto:("caribe ingles" OR "america do sul" OR "caribe" OR "america central"))  w:((mh:(buba)) OR (mj:(pian)) OR (tw:("treponematosis endemicas"))) AND (instance:"regional") AND ( db:("PAHO") AND pais_assunto:("caribe" OR "haiti" OR "america do sul" OR "america do norte" OR "america central" OR "caribe ingles")) |
