# Supplementary material for: Epidemiological situation of yaws in the Americas: A systematic review in the context of a regional elimination goal
Source: PLoS Negl Trop Dis. 2019 Feb 25;13(2):e0007125. doi: 10.1371/journal.pntd.0007125 (PMC6405159; doi:10.1371/journal.pntd.0007125)
Supplement: S1 Fig — (DOCX) [file pntd.0007125.s003.docx]

**PRISMA 2009 Flow Diagram**

####

Studies included in qualitative synthesis
(n = 75)

Full-text articles excluded, with reasons
(n = 148)

- Participants (n = 85)
- Intervention (n = 2)
- Outcome (n = 2)
- Design (n = 1)
- Region (n = 29)
- Not human (n = 1)
- Duplicate (n = 28)

Full-text articles assessed for eligibility
(n = 223)

Records excluded
(n = 249)

Records screened
(n = 472)

Records after duplicates removed
(n = 472)

Additional records identified through other sources
(n = 5)

## Identification

## Eligibility

## Included

## Screening

Records identified through database searching
(n = 674)
